# Adolescents’ Engagement in Ethnic Harassment: Prejudiced Beliefs in Social Networks and Classroom Ethnic Diversity

**DOI:** 10.1007/s10964-017-0795-0

**Published:** 2018-01-02

**Authors:** Sevgi Bayram Özdemir, Shuyan Sun, Liliia Korol, Metin Özdemir, Håkan Stattin

**Affiliations:** 1 Center for Developmental Research, Örebro University, Örebro, Sweden; 2 University of Maryland, Baltimore County, Baltimore, MD USA; 3 National University of Ostroh Academy, Ostroh, Ukraine

## Abstract

Research on ethnic victimization to date has done little to identify the reasons why adolescents victimize their peers due to their ethnic background. To address this limitation, we examined: (1) the extent to which prejudiced attitudes within adolescents’ close and larger social networks determine their engagement in ethnic harassment, and (2) the extent to which classroom ethnic diversity plays a role in any such link. Our sample included 902 Swedish adolescents (*M*_*age*_ = 14.40, SD = .95; 50.3% girls). We found that Swedish adolescents who held negative attitudes toward immigrants or who were surrounded by prejudiced peers were more likely to be involved in ethnic harassment, particularly in classrooms with high ethnic diversity. Adolescents in classrooms with a high anti-immigrant climate were more likely to harass their immigrant peers. These findings suggest that prejudiced beliefs in youth social networks put young people at risk of engaging in ethnic harassment, particularly in ethnically diverse classrooms.

## Introduction

Promoting harmonious inter-ethnic relationships is a new challenge for schools with increasing ethnic diversity in immigrant-receiving countries, including Sweden, but there are barriers to meeting this challenge. A number of studies across cultures have shown that native youth tend not to make friends with peers of immigrant background (Özdemir et al. 2017; Strohmeier and Spiel 2003), and that immigrant youth are exposed to negative treatment at school. For instance, Plenty and Jonsson (2017) reported that immigrant youth in Sweden, especially those from non-European countries, were more likely to be isolated and socially rejected. Similarly, a recent large-scale study (*N* = 3305) in the U.S. found that 12% of the students considered (from grades 5 through 12) reported being targeted by bullies due to their ethnic background (Mendez et al. 2016). Further, a study in the Netherlands found that about 42% of ethnic minority children mentioned being victims of racist name-calling in school, and about 30% had experience of ethnic exclusion (Verkuyten and Thijs 2002). In the long-term, such negative experiences may influence whether and how immigrant children and youth are integrated into the mainstream society as they grow up. Thus, systematic action, informed by scientific evidence, to counteract the problem is urgently needed. In the present study, we aim to tap into this issue by investigating the risk factors that lead youth to victimize or harass their immigrant peers at schools in Sweden.

### Negative Consequences of Ethnic Victimization

A growing body of research has shown that ethnicity-based harassment experiences have negative consequences for youth’s psychological functioning, such as negative self-perception (Bayram Özdemir and Stattin 2014), elevated anxiety, depressive symptoms, and increased stress (Hoglund and Hosan 2013; McKenney et al. 2006; Mendez et al. 2016; Volk et al. 2006). Further, being exposed to ethnic harassment also impacts immigrant adolescents’ school adjustment, such that ethnically harassed youth often have low school satisfaction and a high anticipation of failure. And, they also perceive their relationships with teachers as negative. In turn, these young people show higher rates of truancy (Bayram Özdemir and Stattin 2014). Recent studies have also shown that ethnic harassment experiences pave the way for engagement in problem behaviors (Bayram Özdemir et al. 2017; McKenney et al. 2006; Volk et al. 2006) and separation from the mainstream society (Bayram Özdemir et al. 2017). Specifically, a recent study of immigrant youth in Sweden showed that exposure to ethnic harassment triggered engagement in violent behaviors, such as threatening others verbally, engaging in street fights, and damaging property, even after controlling for the effect of youth’s general experience of harassment (Bayram Özdemir et al. 2017). These findings suggest that exposure to ethnic harassment constitutes a unique risk for engagement in violent behaviors among immigrant youth. Altogether, prior research has provided substantial evidence that ethnic victimization or harassment has harmful consequences for immigrant youth’s psychosocial functioning and school adjustment.

### What Makes Adolescents Harass their Immigrant Peers?

Most of the research on ethnic victimization and harassment to date has adopted a victim perspective, focusing on the consequences of victimization. By contrast, far less attention has been paid to investigating the problem from a perpetrator perspective. It is still unclear what provokes (or restrains) young people with regard to harassing their immigrant peers due to their ethnic background. To our knowledge, there are only two relevant empirical studies in the literature. The first was conducted in Sweden (Bayram Özdemir et al. 2016), and showed that Swedish youth with negative attitudes toward immigrants were more likely to harass their immigrant peers over time, especially if they had impulsive tendencies and engaged in violent behaviors. Bayram Özdemir and colleagues (2016) argued that prejudiced youth with impulsive traits may have difficulties in regulating their negative thoughts and emotions with regard to immigrants, and react coercively without evaluating the effects of their actions on their immigrant peers. The second study was conducted in Canada (Larochette et al. 2010), and reported that boys and youth who were victimized by their peers were more likely to engage in ethnicity-based bullying at school. In this study, it was also found that, when youth attended schools where there were more teachers of a different ethnic background *and* where the teachers treated students more fairly, youth were less likely to engage in ethnic bullying. These two studies primarily focused on perpetrators’ individual characteristics (e.g., gender, views about immigrants, impulsive traits, and victimization experiences) in order to explain why some young people engage in ethnic harassment. Thus, there is limited knowledge regarding whether the social context at school plays a role in terms of youth’s coercive behaviors toward their immigrant peers.

### The Role of Prejudiced Beliefs within Adolescents’ Social Networks

Social norms theory (Perkins and Berkowitz 1986) postulates that people’s perceptions of contextually bound social norms influence their attitudes and behaviors. People generally display behaviors that are in line with the social norms of the context, and try to avoid contradicting group norms so as to avoid social sanctions. In line with this theory, a growing body of research emphasizes that social norms in peer settings determine how children think about immigrant peers (Miklikowska 2017; Nesdale et al. 2005), and also how they interact with them (Tropp et al. 2016; Titzmann et al. 2015). For instance, in an experimental study, Nesdale and colleagues (2005) reported that children were more likely to perceive member of other groups as a threat if their peers had negative attitudes toward out-group members. Further, Titzmann and colleagues (2015) showed that German adolescents were more likely to make friends with immigrants and maintain their friendships over time when their peers had favorable attitudes toward inter-ethnic relationships. Similarly, a recent study in the U.S. showed that adolescents became more comfortable and interested in forming cross-ethnic friendships if they felt that such relationships would be acceptable in their peer groups (Tropp et al. 2016).

Social norms in peer and classroom settings may also affect whether native youth act coercively toward their immigrant peers. The existing literature focusing on group norms and engagement in problem behaviors among adolescents provide evidence of the presence of such coercive action. For instance, Salmivalli and Voeten (2004) showed that pre-adolescents engaged in less bullying behavior in classrooms where the students as a group held strong anti-bullying attitudes. Similarly, Pozzoli and colleagues (2012) found that adolescents were more likely to defend their victimized peers when they perceived bullying to be unacceptable in their classrooms. In line with these findings, it can be argued that native adolescents are more likely to manifest coercive behaviors toward their immigrant peers if they are in a social context where a majority of their peers hold negative attitudes toward immigrants. On the other hand, the possibility of negative evaluations may demotivate youth from displaying aggressive reactions toward their immigrant peers in a social context where positive attitudes toward immigrants are salient. To our knowledge, there is an absence of research on whether group norms impact on youth’s engagement in ethnic harassment. To address this gap in knowledge, we aim to examine the extent to which prejudiced beliefs within adolescents’ close social network (i.e., their best friends) and larger social network (i.e., the classroom setting) influence their engagement in ethnic harassment. We targeted adolescents in this study because young people form their identities on the basis of social categories such as ethnicity and nationality during this developmental period (Tajfel and Turner 1986), and develop ideas about other groups. Further, peer acceptance and conformity become salient in adolescence, and adolescents are sensitive to messages given within their own peer network (Brown and Larson 2009).

### The Role of Classroom Ethnic Diversity

The existing literature on the role of classroom ethnic composition and diversity in the development of negative interactions between native and immigrant youth relies on two theoretical orientations with contradictory conclusions. The first group of studies relies on the premises of the power imbalance theory (Graham 2006; Juvonen et al. 2006). The theory implies that students in ethnically heterogeneous classrooms share a balance of social power. However, as classrooms become more ethnically homogenous, asymmetric power relations arise. Immigrant or minority adolescents often have less numerical or social power and lack support compared with their native peers in such settings. This situation puts them at greater risk of experiencing negative peer treatments. For instance, in a recent study in Sweden, Plenty and Jonsson (2017) showed that immigrant youth in immigrant-sparse classrooms were more likely to be rejected, isolated, and victimized than those in immigrant-dense classrooms. There is a similar finding from another study (Hjern et al. 2013), which used data from a national survey focusing on 15-year-old children in Sweden (*N* = 76 229). Specifically, Hjern and colleagues (2013) showed that first-generation immigrant children in Swedish-dominant schools (particularly those of African and Middle Eastern ethnic backgrounds) were more exposed to bullying than those in immigrant-dense schools. Also, Agirdag and colleagues (2011) showed that immigrant youth attending schools with a higher ethnic minority concentration experienced less peer victimization than those attending schools with fewer non-natives. Taken together, the studies using power imbalance theory suggest that students of immigrant background are more likely to be victimized in native-dominant or ethnically homogenous settings.

The second group of studies relies on the ethnic-group competition theory (Coenders et al. 2004). This theory suggests that individuals from the mainstream society may perceive an increasing number of people of ethnic minority as a threat to the very existence of their cultural norms and values. Supporting this theory, a cross-national study in Europe showed that people from countries with a higher percentage of non-European immigrants perceived immigrants as a burden in terms of social stability and economic welfare (Scheepers et al. 2002). The ethnic-group competition theory has also been used to explain the relationships between native and immigrant youth in different school contexts (Vervoort et al. 2010; Vervoort et al. 2011). For instance, Vervoort and colleagues investigated the relation between classroom ethnic composition (defined as the proportion of students of non-Western ethnic minority in class) and adolescents’ out-group attitudes and engagement in victimization. They found that Dutch adolescents in classrooms with a large proportion of ethnic minority students had more negative out-group attitudes (Vervoort et al. 2011), and engaged in more peer victimization (Vervoort et al. 2010), than those in classrooms with no or a small proportion of ethnic minority students. Together, these studies suggest that victimization of immigrant students may be more prevalent in ethnically-mixed classrooms than immigrant-sparse classrooms.

Classroom ethnic composition or diversity may also influence the extent to which native youth are influenced by the norms in their social context and how they behave toward their immigrant peers. To our knowledge, this issue has not been investigated before. To address this gap in knowledge, we examine whether classroom ethnic diversity moderates the effects of prejudiced beliefs within youth’s close and larger social networks on their engagement in ethnic harassment. Two competing hypotheses can be proposed here. First, as highlighted in the power imbalance theory, native adolescents often have greater numerical or social power in ethnically homogenous classrooms (Graham 2006; Juvonen et al. 2006). This situation might create a suitable context for youth who are surrounded by prejudiced peers or classmates to act out. Specifically, these young people may perceive the numerical shortage of immigrants as an advantage, and in turn consider their immigrant peers as easy targets to victimize. Accordingly, there may be a tendency among native youth to act coercively toward their immigrant peers in ethnically homogenous classrooms. Alternatively, in line with the ethnic competition theory (Coenders et al. 2004), it can be argued that youth who hang out with prejudiced peers or are in classes with high anti-immigrant attitudes may come to perceive immigrant peers as a threat to their social power as the classroom context becomes heterogeneous. Thus, in ethnically diverse classrooms, they may be more inclined to engage in ethnic harassment, so as to diminish the social threat or to acquire social dominance.

### The Role of Adolescent Gender

A large body of literature demonstrates that adolescent boys tend to engage in more deviant and aggressive behaviors than girls (e.g., Leadbeater et al. 1999; Thijs et al. 2015). And, there are similar findings in studies focusing on ethnicity-based bullying and harassment (e.g., Bayram Özdemir et al. 2016; Larochette et al. 2010). Prior research also suggests that the social context may influence youth’s engagement in problem behaviors differently across the genders. For instance, Salmivalli and colleagues showed that girls, compared with boys, were more likely to engage in bullying when they were in a classroom where pro-bullying attitudes were prevalent (Salmivalli and Voeten 2004) and when their peers had a greater tendency to engage in bullying (Salmivalli et al. 1998). Such gender differences may also be reflected in the way adolescents harass peers due to their immigrant background. Thus, in this study, we examined whether the boys or girls in our sample were influenced by prejudiced beliefs in their close and larger social networks, and became more engaged in ethnic harassment. Relying on previous research on bullying, we expected that girls would be more influenced by their peers’ attitudes toward immigrants, and prejudiced beliefs within their classrooms, than boys.

## The Current Study

The present study aims to further our understanding of why adolescents engage in ethnicity-based harassment in schools. Specifically, we address two questions. Our first goal was to examine the extent to which prejudiced beliefs within youth’s close social network (i.e., their best friends) and larger social network (i.e., the classroom setting) influence their engagement in ethnic harassment. On the basis of social norms theory (Perkins and Berkowitz 1986) and previous research on group norms (e.g., Nesdale et al. 2005; Salmivalli and Voeten 2004; Tropp et al. 2016), we expected that youth who are in a social context where there are prejudiced beliefs toward immigrants are more likely to engage in ethnic harassment. Our second goal was to examine whether classroom ethnic diversity determines the extent to which youth are influenced by the norms in their social context, and, in turn, harass their immigrant peers. We did not propose any directional pathway because existing theoretical arguments (Coenders et al. 2004; Graham 2006) suggest that classroom ethnic diversity might act as either a buffer or a risk factor.

## Methods

### Participants

The sample for the present study comes from a longitudinal investigation—the Seven School Study—which was conducted by Håkan Stattin and Margaret Kerr. The main aim of the Seven School Study was to understand youth’s experiences inside and outside school, and their relationships with their parents, peers, and teachers. It was conducted in seven schools in Örebro, which is a medium-sized town in Sweden. Neighborhood characteristics were considered in selection of the schools. Specifically, we took into account the socio-economic characteristics of the neighborhoods in Örebro in order to have schools with a wide range of native-to-immigrant youth ratios. In each school, students from 7th to 9th grades were targeted, and they were assessed every year until they graduated from secondary school. The target sample included 1654 adolescents. Of the target sample, 89% participated in the study. Among the participating adolescents (*N* = 1485), 63% of them were Swedish (i.e., those whose parents were born in Sweden or another Nordic country, including Finland, Norway, and Denmark). The rest (37%) were either mixed or immigrant adolescents whose parents had migrated to Sweden, from different countries, representing regions in the Middle East (e.g., Egypt, Iran, Iraq, Lebanon, and Syria), Africa (e.g., Eritrea, Gambia, Somalia, and Tunisia), Asia (e.g., India, Malaysia, Thailand, and Vietnam), South and Central America (e.g., Colombia, El Salvador, and Uruguay), Europe (e.g., Greece, Poland, and Spain), Russia, and former Yugoslavia (e.g., Bosnia, Kosovo, and Serbia). The analytical sample for the current study comprised Swedish adolescents only (*N* = 902; *M*_*age*_ = 14.40, SD = .95; 50.3% girls). A majority of the adolescents were from two-parent households (64%) and had employed parents (88% of the mothers, and 93% of the fathers).

## Procedure

Data collection was held during regular class hours, and overseen by trained research assistants. Students were informed about the goals of the study and ethical issues including voluntary participation, privacy, and data confidentiality. Only students who were willing to participate and whose parents did not decline their children’s participation took part in the study. The questionnaires were administered in Swedish only.

### Measures

#### Attitudes toward immigrants

We used an eight-item scale to assess adolescents’ attitudes toward immigrants (van Zalk et al. 2013). The sample items included: “Immigrants often come here just to take advantage of welfare in Sweden” and “Immigrants often take jobs from people who are born in Sweden”. The adolescents were asked to rate each item on a 4-point scale, ranging from “1” (Don’t agree at all) to “4” (Agree completely). Their responses to the items were averaged to create scale scores. Higher scores indicated high negative attitudes toward immigrants. Inter-item reliability was .80.

#### Best friends’ attitudes toward immigrants

The adolescents were first asked to nominate three peers in their school whom they considered very important in their lives, and whom they often talked to, spent time with, and did things together with. Eighty percent of the adolescents reported having three friends, and only 4% of the adolescents did not nominate any friend. Using a software program (PERL), we were able to restructure the data (i.e., place both youth and their best friends’ answers to the survey questions in the same row), and thereby assess each friend’s attitudes toward immigrants. Then, we averaged the attitudes of each friend to create a measure of friends’ negative attitudes toward immigrants.

#### Classroom-level attitudes toward immigrants

Classroom-level attitudes toward immigrants were measured by averaging the negative attitudes of all students, including both Swedish and immigrant youth, in each classroom.

#### Classroom ethnic diversity

We created an ethnic diversity index for each classroom using a formula developed by Simpson (1949). The ethnic-diversity index captures both the number of different ethnic groups and the relative representation of each group in each classroom. The formula reads: $D_c = 1 - \mathop {\sum }\nolimits_{i = 1}^g p_i^2$, where D_*C*_ represents the ethnic diversity of a classroom, and *p*_*i*_ is the proportion of students in the classroom who belong to ethnic group *i*. Scores can range from 0 to 1, with higher numbers reflecting greater ethnic diversity. In the present study, the mean ethnic diversity across classrooms was .48. In calculation of the index, we used the data that were reported by the adolescents on their parents’ country of birth. In the present study “classroom” was defined as the setting where students take basic courses together (a minority of the courses are elective and may contain peers from other classes). The average classroom size was about 23 in the target sample, and the participation rate was over 80% in majority of the classrooms.

#### Ethnic harassment

A single item was used to measure how often the adolescents had harassed their peers on ground of their ethnic background (Bayram Özdemir et al. 2016). The item was: “Have you said nasty things to anyone in school this semester, simply because that person was an immigrant?” The young people answered this question on a scale ranging from “1” (No, it has not happened) to “4” (Yes, it has happened several times a week). 91% of the participants reported a score of 1, resulting in a non-normal distribution (Skewness 4.21, SE = 0.08; Kurtosis = 19.90, SE = 0.17). Therefore, a linear multilevel model that requires normality was not appropriate. To deal with the non-normal distribution of ethnic harassment, the adolescents’ responses were recoded as 0 “No engagement in harassment” and 1 “Engagement in ethnic harassment at least once” so that a generalized linear mixed effects model could be applied. This item has been used previously to measure youth’s engagement in ethnic harassment, and evidence for the criterion validity and discriminant validity has been provided (Bayram Özdemir et al. 2016).

#### Perceived socio-economic status

We used the following three items to assess how the adolescents perceived their socio-economic status. The questions and response options were: “Have you not been able to do something with your friends this semester because you could not afford it?” (1 = *Yes, several times* to 3 = *No*); “If you want a thing that costs a lot of money (for example, a computer, skateboard, cell phone), can your parents afford to buy it if they think you need it?” (1 = *No, probably not* to 3 = *Yes, probably*); and “What is the financial situation in your family?” (1 = *My parents often complain about not having enough money* to 3 = *My parents never complain about not having enough money*). These items have previously been used to measure adolescents’ perception of family socio-economic status (Bayram Özdemir et al. 2017; Svensson et al. 2012). The item-total correlations between these three items were satisfactory, ranging from .35 to .46. We created a perceived SES index based on the average score of the standardized values of the three items. Perceived SES was controlled for in all analyses because several studies have shown that youth of low socio-economic status are at risk of engagement in problem behaviors, including bullying (Jansen et al. 2011; see Tippett and Wolke 2014, for a review).

### Data Analysis

The observations in the current data were clustered by classroom. Thus, we first examined how much of the variation in the outcome variable was between classrooms in order to see if there was any need to use a multilevel regression model (Hox 2002). We estimated a generalized linear mixed-effects model with no predictor, which allowed us to partition the variation in engagement in ethnic harassment within and between classrooms (Heck et al. 2013). The results indicated that about 10% of the variation in engagement in ethnic harassment was between classrooms (design effect = 2.19), suggesting that use of a multilevel model was necessary for the current analysis (Muthen and Satorra 1995). Accordingly, we estimated generalized linear mixed-effects models to test our research questions. In all models, youth’s SES was controlled for. Generalized linear mixed-effects models were analyzed using Mixed Models in SPSS.

## Results

### Descriptive Statistics and Preliminary Analyses

Descriptive statistics and bivariate correlations between the study variables are presented in Table 1. Boys were more likely to engage in ethnic harassment than girls (phi-coefficient = −.18, *p* < .001). As expected, there was a positive correlation between youth’s negative attitudes toward immigrants and their engagement in ethnic harassment, such that youth who engaged in ethnic harassment had statistically higher negative attitudes than those who did not engage in ethnic harassment. Moreover, prejudiced beliefs within youth’s close social network (i.e., their best friends) and larger social network (i.e., the classroom setting) were positively associated with negative attitudes toward immigrants.

**Table 1:** Descriptive statistics and bivariate correlations among the study variables

	(1)	(2)	(3)	(4)	(5)	*Min*	*Max*	*M*	SD
(1) Socioeconomic status						−2.39	0.88	−0.02	0.76
(2) Gender	−.01					-	-	-	-
(3) Adolescents’ negative attitudes toward immigrants	−.02	−.11**				1.00	4.00	2.37	0.62
(4) Class negative attitudes toward immigrants	−.07*	−.05	.33**			1.84	2.81	2.30	0.20
(5) Friends’ negative attitudes toward immigrants	−.03	−.15**	.29**	.45**		1.00	4.00	2.30	0.48
(6) Ethnic harassment	−.09*	−.18**	.18**	.13**	.10**	0.00	1.00	0.09	0.29

Gender was coded as: “0” Male and “1” Female

*p < .05; **p < .001

### Do Peers’ Prejudiced Beliefs and the Classroom Inter-Ethnic Climate Play a Role in Adolescents' Engagement in Ethnic Harassment?

We used a series of generalized linear mixed-effects models to examine the extent to which best friends’ and class-level attitudes toward immigrants predicted youth’s engagement in ethnic harassment, above and beyond youth’s socio-economic status and own attitudes toward immigrants (see Table 2). In Model 1, SES was the only predictor. The results showed that Swedish youth with low SES were more likely to harass their immigrant peers than those with high SES, *b* *=* −.33, SE *=* .15, *t* = −2.22, *p* *=* .03, OR = .72. In Model 2, youth’s negative attitudes toward immigrants were entered. We found that, after controlling for SES, youth’s negative attitudes toward immigrants significantly predicted their engagement in ethnic harassment, such that, as youth’s negative attitudes increased by 1 unit, the odds of engagement in ethnic harassment rose 2.64 times. In Model 3, friends’ negative attitudes toward immigrants and class-level negative attitudes were entered simultaneously. Class-level negative attitudes significantly predicted youth’s engagement in ethnic harassment, above and beyond their SES and own attitudes, *b* *=* 1.58, SE *=* .77, *t* = 2.05, *p* *=* .04, OR = 4.86. Specifically, as class-level negative attitudes increased by 1 unit, the odds of engagement in ethnic harassment increased 4.86 times. By contrast, friends’ negative attitudes toward immigrants did not significantly predict youth’s engagement in ethnic harassment. Together, these findings suggest that prejudiced beliefs in classroom settings elevate the risk of engagement in ethnic harassment among Swedish youth.

**Table 2:** Logistic regression predicting adolescents’ engagement in ethnic harassment from negative attitudes toward immigrants

	Model 1^a^			Model 2^b^			Model 3^c^		
	*b*	SE	*OR*	*b*	SE	*OR*	*b*	SE	*OR*
Intercept	−2.37**	0.15	0.09	−4.83**	0.56	0.01	−8.24**	1.60	0.00
SES	−0.33*	0.15	0.72	−0.34*	0.15	0.71	−0.31	0.16	0.73
Adolescents’ negative attitudes toward immigrants				0.97**	0.20	2.64	0.78**	0.22	2.18
Friends’ negative attitudes toward immigrants							0.10	0.29	1.10
Class negative attitudes toward immigrants							1.58*	0.77	4.86

^a^ Model 1 summary: AIC = 4509.59, BIC = 4514.33, level-2 variance component = 0.37, SE = 0.19, *p* = .06

^b^ Model 2 summary: AIC = 4549.17, BIC = 4553.90, level-2 variance component = 0.26, SE = 0.18, *p* = .16

^c^ Model 3 summary: We initially estimated the level-2 variance component in the model; however, the Hessian matrix was not positive definite due to lack of variance between classrooms. Therefore, we set the level-2 variance component at zero and re-estimated the model, for which AIC = 450.94, BIC = 474.26.

*p *<* .05; **p *<* .001

### Does Classroom Ethnic Diversity Play a Role in Adolescents' Engagement in Ethnic Harassment?

We estimated three separate generalized linear mixed-effects models. In the first model, we investigated whether classroom ethnic diversity moderated the relation between youth’s attitudes toward immigrants and their engagement in ethnic harassment. In the second and third models, we examined the extent to which classroom ethnic diversity moderated the associations between prejudiced beliefs within youth’s close and larger social networks and their engagement in ethnic harassment. In these models, youth’s SES and negative attitudes toward immigrants were controlled for. Classroom ethnic diversity and each of the negative attitude variables (i.e., youth’s own attitudes, best friends’ attitudes, and class-level attitudes) were mean-centered. Then, interaction terms between classroom ethnic diversity and the attitude variables were created.

**Fig. 1:**
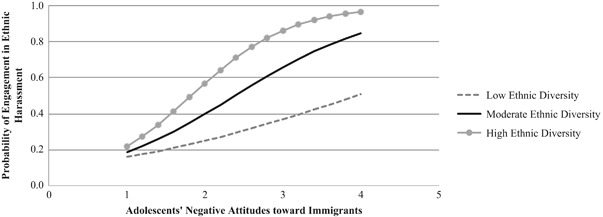
The interaction between adolescents’ negative attitudes toward immigrants and classroom ethnic diversity in predicting adolescents’ engagement in ethnic harassment. Probability values were estimated when SES was set at its mean of −0.02

The results from the first model (see Table 3) showed that there was a statistically significant interaction between youth’s negative attitudes toward immigrants and classroom ethnic diversity (*b* = 2.11, SE = 0.92, *p* = .02, OR = 8.23). Youth’s own negative attitudes toward immigrants significantly predicted engagement in ethnic harassment in all three types of classrooms, but the odds were higher for students in classrooms with high ethnic diversity. Specifically, at one SD below the mean of classroom ethnic diversity, as youth’s negative attitude increased by one unit, the odds of engagement in ethnic harassment increased by 1.77 (*b* = 0.57, SE = 0.27, *p* = .03). At the mean level of classroom ethnic diversity, as youth’s negative attitude increased by one unit, the odds of engagement in ethnic harassment increased by 2.90 (*b* = 1.07, SE = 0.21, *p* < .001). At one SD above the mean of classroom ethnic diversity, as youth’s negative attitude increased by one unit, the odds of engagement in ethnic harassment increased by 4.76 (*b* = 1.56, SE = 0.33, *p* < .001). The moderating effect of classroom ethnic diversity on the relation between youth’s attitudes and ethnic harassment is illustrated in Fig. 1. Together, these findings suggest that the youth with negative attitudes toward immigrants engaged in more ethnic harassment as classroom ethnic diversity increased.

**Table 3:** The moderating role of classroom ethnic diversity in predicting adolescents’ engagement in ethnic harassment from adolescents’ attitudes toward immigrants

	*b*	SE	*t*	*p*	*OR*
Intercept	−2.56	0.15	−17.19	<.001	0.08
SES	−0.39	0.15	−2.51	.01	0.68
Adolescents’ negative attitudes toward immigrants	1.07	0.21	5.16	<.001	2.90
Classroom ethnic diversity	−1.27	0.66	−1.91	.06	0.28
Adolescents’ negative attitudes * Classroom ethnic diversity	2.11	0.92	2.28	.02	8.23

We initially estimated the level-2 variance component in the model; however, the Hessian matrix was not positive definite due to lack of variance between classrooms. Therefore, we set the level-2 variance component at zero and re-estimated the model, for which AIC = 462.99, BIC = 486.57

**Fig. 2:**
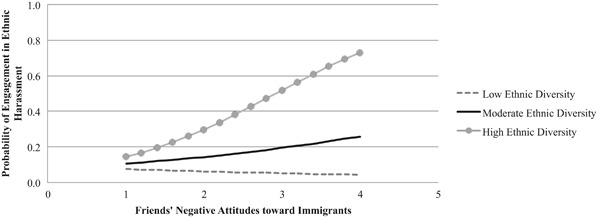
The interaction between friends’ negative attitudes toward immigrants and classroom ethnic diversity in predicting adolescents’ engagement in ethnic harassment. Probability values were estimated when SES was set at its mean of −0.02, and youth’s attitudes toward immigrants were set at their mean of 2.37

The results from the second model (see Table 4) showed that there was a statistically significant interaction between friends’ negative attitudes toward immigrants and classroom ethnic diversity. Specifically, we found that friends’ negative attitudes did not predict youth’s engagement in ethnic harassment at one SD below the mean of classroom ethnic diversity (*b* = −0.19, SE = 0.38, *p* = .61, OR = 0.82). Also, at the mean of classroom ethnic diversity, friends’ negative attitudes did not predict youth’s engagement in ethnic harassment (*b* = 0.37, SE = 0.27, *p* = .17, OR = 1.44). However, at one SD above the mean of classroom ethnic diversity, friends’ negative attitudes significantly predicted youth’s engagement in ethnic harassment, such that as friends’ negative attitudes increased by 1 unit, the odds of engagement in ethnic harassment increased by 2.53 (*b* = 0.93, SE = 0.39, *p* = .02, OR = 2.53). The interaction effect is illustrated in Fig. 2. Together, these findings suggest that best friends’ prejudiced beliefs have more influence on youth’s engagement in ethnic harassment in classrooms with high ethnic diversity.

**Table 4:** The moderating role of classroom ethnic diversity in predicting adolescents’ engagement in ethnic harassment from friends’ attitudes toward immigrants

	*b*	SE	*t*	*p*	*OR*
Intercept	−4.63	0.58	−7.98	<.001	0.01
SES	−0.36	0.16	−2.25	.03	0.70
Adolescents’ negative attitudes toward immigrants	0.89	0.22	4.11	<.001	2.43
Friends’ negative attitudes toward immigrants	0.37	0.27	1.36	.17	1.44
Classroom ethnic diversity	−0.82	0.60	−1.36	.18	0.44
Friends’ negative attitudes * Classroom ethnic diversity	2.38	1.15	2.07	.04	10.85

We initially estimated the level-2 variance component in the model; however, the Hessian matrix was not positive definite due to lack of variance between classrooms. Therefore, we set the level-2 variance component at zero and re-estimated the model, for which AIC = 452.29, BIC = 480.26.

The results from the third model showed that the interaction between class-level attitudes toward immigrants and classroom ethnic diversity was not statistically significant. Therefore, the model was re-analyzed without the interaction term. As shown in Table 5, the main effect of class-level negative attitudes on youth’s engagement in ethnic harassment was statistically significant after controlling for the effects of SES, youth’s negative attitudes, and classroom ethnic diversity. Specifically, as class-level negative attitudes increased by 1 unit, the odds of engagement in ethnic harassment increased by 4.03. In sum, these findings suggest that a negative inter-ethnic climate in classrooms paves the way for engagement in ethnic harassment among youth, regardless of classroom ethnic diversity.

### Does Youth Gender Play a Role in How Youth are Influenced by Prejudiced Beliefs in Their Social Environment?

We estimated two separate models to examine the moderating roles played by youth gender in the relations between prejudiced beliefs within youth’s close and larger social networks and their engagement in ethnic harassment. Gender significantly predicted engagement in ethnic harassment, such that boys were more at risk than girls. By contrast, gender did not act as a significant moderator. Together, these findings suggest that boys and girls are influenced similarly by prejudiced beliefs in their social networks.

**Table 5:** The role of classroom ethnic diversity in predicting adolescents’ engagement in ethnic harassment from class negative attitudes toward immigrants

	*b*	SE	*t*	*p*	*OR*
Intercept	−7.74	1.70	−4.56	<.001	0.00
SES	−0.34	0.15	−2.23	.03	0.71
Adolescents’ negative attitudes toward immigrants	0.86	0.21	4.10	<.001	2.36
Class negative attitudes toward immigrants	1.40	0.72	1.94	.05	4.04
Classroom ethnic diversity	−0.10	0.56	−0.19	.85	0.90

We initially estimated the level-2 variance component in the model; however, the Hessian matrix was not positive definite due to lack of variance between classrooms. Therefore, we set the level-2 variance component at zero and re-estimated the model, for which AIC = 465.50, BIC = 489.09.

## Discussion

The majority of existing research on ethnic victimization and harassment has adopted a victim perspective, highlighting the detrimental effects of victimhood on immigrant youth’s adjustment into a host society (e.g., Bayram Özdemir and Stattin 2014; McKenney et al. 2006; Verkuyten and Thijs 2002). Yet, relatively little attention has been paid to understanding the problem from the perpetrator’s perspective (Bayram et al. 2016; Larochette et al. 2010). The present study aimed to address this gap in knowledge by investigating the risk factors that lead native adolescents to harass their immigrant peers at school in Sweden. Specifically, we examined the extent to which prejudiced beliefs toward immigrants within youth’s close social network (i.e., their best friends) and larger social network (i.e., the classroom setting) influence their involvement in ethnic harassment. Further, we tested whether classroom ethnic diversity determines the extent to which youth are influenced by the norms in their social context, and, in turn, harass their immigrant peers.

Our findings show that Swedish youth with negative attitudes toward immigrants engaged in more ethnic harassment as classroom ethnic diversity increased. Consistent with the premises of ethnic-group competition theory (Coenders et al. 2004), the presence of a higher proportion of students with immigrant background in class may be perceived by prejudiced adolescents as a threat to their dominant status. Thus, these adolescents may be more inclined to harass their immigrant peers to maintain their social dominance or reduce the perceived threat. Alternatively, it is possible that adolescents with pre-existing negative ideas about out-group members have greater opportunities to find a victim in these classrooms, and, in turn, act out consistently with their attitudes. In line with this interpretation, previous research has shown that greater ethnic heterogeneity in class can lead to increased tension between immigrant and non-immigrant students, and result in more negative out-group attitudes (Vervoort et al. 2011) and increased aggressive behaviors, including physical fighting and bullying (Vervoort et al. 2010; Walsh et al. 2016). Overall, our findings indicate that youth’s prejudiced out-group perceptions form a motivational basis for their engagement in coercive behaviors toward their immigrant peers. Importantly, such problematic behaviors are most likely to be observed in ethnically diverse classrooms. This finding suggests that promoting inter-ethnic relationships requires not only mixing adolescents of different ethnic backgrounds in schools but also fostering students’ positive views about one another.

Importantly, the present research draws attention to the importance of norms in adolescents’ social networks in determining adolescents’ engagement in ethnic harassment. Supporting the premises of social norms theory (Perkins and Berkowitz 1986) and the findings of previous studies (Nesdale et al. 2005; Tropp et al. 2016), we found that whom adolescents hang out in school with matters for their involvement in ethnic harassment. Specifically, we showed that the Swedish adolescents who were surrounded by prejudiced friends were more likely to act aggressively toward their immigrant peers in classrooms with high ethnic diversity. A possible explanation for this finding is that adolescents with prejudiced friends may be more exposed to negative portrayals of immigrants in their social interactions in schools, and such exposures may fuel their anti-immigrant attitudes (Miklikowska 2017). In addition, socializing with prejudiced friends may amplify perceptions of the threat of social dominance among adolescents, especially with a large number of immigrants in class. As a result, these adolescents may become more motivated to act aggressively toward their immigrant peers, both to overcome their negative feelings and to conform to the norms that are salient in their close social network (Nesdale and Dalton 2011).

We should also note that, in the present study, the profile of immigrant youth in ethnically diverse classrooms was different from those in classrooms with low ethnic diversity. Specifically, a majority of the immigrant youth in ethnically diverse classrooms was from Middle Eastern and African countries (e.g., Iraq, Syria, Somalia); they are regarded as visible immigrants and experience integration problems in Sweden (Hjern et al. 2013). On the other hand, the immigrant students in the classrooms with low ethnic diversity were mostly from the former Yugoslavian countries (e.g., Bosnia), and are part of one of the well-integrated immigrant groups. Such differences in the profiles of immigrants across high and low ethnically diverse classrooms may be one of the potential reasons why native youth engage in more ethnic harassment in ethnically diverse classrooms, especially when they have negative attitudes or when they are surrounded by prejudiced peers. Already existing cultural or religious differences between native and immigrant youth may become more salient and intimidating for native youth in ethnically diverse classrooms. They might experience more cultural clashes, which result in engagement in more coercive behaviors toward their immigrant peers.

A noteworthy conclusion of the present study is that the adolescents who were in classrooms with high anti-immigrant attitudes were more likely to engage in ethnic harassment across all classrooms regardless of the level of ethnic diversity. Importantly, this result held even after we controlled for adolescents’ SES and their own negative attitudes toward immigrants. The finding indicates that being in a larger social network with a high prevalence of prejudiced beliefs toward immigrants is an important risk factor for adolescents’ involvement in ethnic harassment. Classmates may constitute an important reference group of peers, and adolescents may be highly receptive to the information they provide about social realities (Thijs et al. 2010). Thus, adolescents may perceive immigrant peers from the perspective of their classmates, and adopt their attitudes and beliefs, in forming their own personal opinions (Thijs and Verkuyten 2013). Consistent with this line of reasoning, prior research has shown that perceived classroom norms regarding out-group attitudes are positively related to students’ self-attitudes toward out-groups (Gniewosz, and Noack 2008; Thijs and Verkuyten 2013). Applied to the context of the current study, it is possible that Swedish adolescents may internalize anti-immigrant attitudes held by the majority of their classmates, and display coercive behaviors toward their immigrant peers, given the lack of possible negative evaluations of such behaviors in their larger social context.

Consistent with prior research (e.g., Bayram Özdemir et al. 2016; Larochette et al. 2010), our results show that gender significantly predicts involvement in ethnic victimization, implying that boys are more likely to harass their immigrant peers than girls. Yet, our findings reveal that boys and girls are equally likely to engage in ethnic bullying under the influence of prejudiced beliefs in their social context. Thereby, the findings imply that norms endorsed in close and larger social networks play an important role in shaping coercive behaviors toward minority peers, regardless of the gender of the perpetrator.

Several limitations should be considered when interpreting the findings of the current study. First, we focused on ethnic harassment behaviors that were displayed by native youth toward immigrants. However, not only native youth engage in ethnicity-based victimization; immigrant adolescents may also behave coercively toward their native and immigrant peers. In fact, a qualitative study in England showed that Asian children (i.e., Hindu, Indian Muslim, and Pakistani) were not only bullied by their native British peers, but also by other Asian children (Eslea and Mukhtar 2000). In addition, we focused on Swedish youth’s views about immigrants, but did not examine how immigrant youth view Swedish society in general and their Swedish peers in particular. It is possible that immigrant youth’s views about Swedish peers might change when they are exposed to ethnic victimization in school. In fact, a recent study showed that immigrant adolescents in Sweden feel more separated from the society when they are exposed to racist name-calling and discriminatory treatment at school (Bayram Özdemir et al. 2017). Together, a holistic understanding of the precursors and conditions of ethnic harassment requires a detailed examination of inter-ethnic relations, i.e., between members of different ethnic groups. Second, we, largely, did not take the ethnic backgrounds of the immigrant victims into account, but tended to treat them as a homogenous group. In general, we assessed attitudes toward immigrants as a general group of foreigners, and did not differentiate between specific groups. Future research can offer a more in-depth analysis of the mechanisms and conditions that explain when youth are more likely to engage in ethnic bullying by better considering the ethnic backgrounds of victims. Third, the present study was correlational by nature, and the data captured only one time-point. Given the inherent limitations of cross-sectional data, it was not possible to test whether there is a causal link between attitudes toward immigrants and youth’s engagement in ethnic harassment. Fourth, we used a single item to assess native youth’s engagement in verbal harassment of their immigrant peers on the basis of their ethnicity. Although this approach has been adopted in previous studies (e.g., Bayram Özdemir et al. 2016; Larochette et al. 2010), it may not provide in-depth information about the different ways in which youth harass their peers. Young people may engage in various coercive behaviors, including physical coercion, social exclusion and relational victimization, when they relate to immigrants of a similar age. Thus, research that examines different manifestations of ethnic harassment is needed to provide a more comprehensive understanding of why youth victimize their immigrant peers.

## Conclusions

Today’s children and youth are growing up in ethnically diverse settings. Current projections suggest that ethnic diversity, especially in developed countries, will continue to increase, which may give rise to escalation of ethnically motivated aversive encounters between members of different ethnic groups. Both engagement in aggressive acts and exposure to harassment may hamper the well-being and healthy development of adolescents (Bouman et al. 2012; Reijntjes et al. 2010). Thus, there is an urgent need to develop a deeper understanding of the developmental processes, conditions, and consequences of living in increasingly diverse settings. The current study gives deeper insight into the risk factors for youth’s engagement in ethnic harassment. Our findings highlight the importance of accounting for prejudiced beliefs in social context in order to understand what makes native adolescents victimize their immigrant peers. The evidence obtained in the study suggests that youth who hold negative attitudes toward immigrants, or hang out with prejudiced friends, are more likely to get involved in ethnic harassment, particularly in ethnically diverse classes at school. Our results may be of significance for programs that aim to improve inter-ethnic relationships in schools. The present study suggests that creating ethnically heterogeneous classrooms, thereby providing students with greater opportunities for cross-group interactions, may not on its own be sufficient to foster positive inter-ethnic relationships. In fact, to the contrary, ethnically diverse settings may create an arena for the development of inter-ethnic conflicts among some youth. Our study emphasizes that future programs should also address the social aspects of school context (in particular, social norms about diversity in peer groups and classrooms) to tackle negative interactions between adolescents of different ethnic backgrounds, and promote harmonious inter-ethnic relationships in schools.
